# Defining Post-COVID Symptoms (Post-Acute COVID, Long COVID, Persistent Post-COVID): An Integrative Classification

**DOI:** 10.3390/ijerph18052621

**Published:** 2021-03-05

**Authors:** César Fernández-de-las-Peñas, Domingo Palacios-Ceña, Víctor Gómez-Mayordomo, María L. Cuadrado, Lidiane L. Florencio

**Affiliations:** 1 Department of Physical Therapy, Occupational Therapy, Physical Medicine and Rehabilitation, Universidad Rey Juan Carlos (URJC), 28922 Madrid, Spain; lidiane.florencio@urjc.es; 2 Department of Neurology, Hospital Clínico San Carlos, 28040 Madrid, Spain; vicmayordomo@gmail.com (V.G.-M.); mlcuadrado@med.ucm.es (M.L.C.); 3 Department of Medicine, School of Medicine, Universidad Complutense de Madrid, 28040 Madrid, Spain

**Keywords:** COVID-19, long COVID, persistent, symptoms, classification, definition

## Abstract

The pandemic of the coronavirus disease 2019 (COVID-19) has provoked a second pandemic, the “long-haulers”, i.e., individuals presenting with post-COVID symptoms. We propose that to determine the presence of post-COVID symptoms, symptoms should appear after the diagnosis of SARS-CoV-2 infection; however, this situation has some problems due to the fact that not all people infected by SARS-CoV-2 receive such diagnosis. Based on relapsing/remitting nature of post-COVID symptoms, the following integrative classification is proposed: potentially infection related-symptoms (up to 4–5 weeks), acute post-COVID symptoms (from week 5 to week 12), long post-COVID symptoms (from week 12 to week 24), and persistent post-COVID symptoms (lasting more than 24 weeks). The most important topic is to establish the time reference points. The classification also integrates predisposing intrinsic and extrinsic factors and hospitalization data which could promote post-COVID symptoms. The plethora of symptoms affecting multiple systems exhibited by “long-haulers” suggests the presence of different underlying mechanisms.

## 1. Should Post-COVID Symptoms Be Considered a New “Syndrome”?

In 2020, the world suffered the most dramatic and catastrophic experience of the last century due to the pandemic of the coronavirus disease 2019 (COVID-19) caused by SARS-CoV-2. In fact, there has been an unprecedented explosion of research in this area. However, a second pandemic has dramatically emerged, i.e., people suffering from symptoms after SARS-CoV-2 infection (“long-haulers”) [1]. Since millions of people have been infected and more will continue to be infected, the number of “long-haulers” is dramatically increasing [2]. Therefore, healthcare professionals and researchers need to identify, classify, and understand the sequelae of COVID-19. Thus, the first step should be an agreement on the terminology [3]. This is a key issue since “long COVID” is considered the first illness term to have been collectively coined by patients themselves through social media [4]. This commentary presents three topics related to post-COVID symptoms, i.e., definition, timeframe, and syndrome [5], which need clarification, and proposes an integrative classification for this potential new “syndrome”.

Although the literature on post-COVID symptoms is still in its earlier stages, “long-haulers” report a plethora of symptoms affecting different systems: neurocognitive post-COVID (brain fog, dizziness, loss of attention, confusion), autonomic post-COVID (chest pain, tachycardia, palpitations), gastrointestinal post-COVID (diarrhea, abdominal pain, vomiting), respiratory post-COVID (general fatigue, dyspnea, cough, throat pain), musculoskeletal post-COVID (myalgias, arthralgias), psychological-related post-COVID (post-traumatic stress disorder, anxiety, depression, insomnia), and other manifestations (ageusia, anosmia, parosmia, skin rashes). In fact, most published studies to date on post-COVID symptoms have found that 50–70% of hospitalized patients exhibit several post-COVID symptoms up to 3 months after hospital discharge [6,7,8,9,10,11]. Data from non-hospitalized COVID-19 patients are scarce and reveal that 50–75% are symptom-free one month after symptom onset [12,13]. It would be expected that post-COVID symptoms will be different between hospitalized and non-hospitalized patients, but this assumption needs to be confirmed in future studies.

The British Medical Association defines a syndrome “as a set of medical signs and symptoms which are correlated with each other and associated with a particular disease [14]. Therefore, the first question arising from this discussion is: should this broad set of post-COVID symptoms be considered a new “syndrome”?

## 2. How Should Post-COVID Symptoms Be Defined?

“Long COVID” is a general term used for people who have recovered from COVID-19 but still exhibit symptoms for far longer than would be expected [15]. Another definition consists of “not recovering several weeks or months following the start of symptoms that were suggestive of COVID-19, regardless individuals were tested or not” [16]. The Guideline published by the National Institute for Health and Care Excellence (NICE), the Scottish Intercollegiate Guidelines Network, and the Royal College of General Practitioners has defined long COVID as “signs and symptoms developed during or following a disease consistent with COVID-19 and which continue for more than four weeks but they are not explained by alternative diagnoses” [17]. Therefore, the first question to discuss is as follows: is a positive test for SARS-CoV-2 or the presence of positive antibodies a prerequisite for the diagnosis? This subject has already been discussed by Raveendran, who proposed three long COVID categories [18]:

- Confirmed (positive diagnosis of SARS-CoV-2 with real-time reverse transcription-polymerase chain reaction [RT/PCR] and/or positive SARS-CoV-2 antibodies testing);
- Probable (symptoms consistent with COVID-19, with negative RT-PCR and/or antibody test, with or without positive radiological signs but WITH contact with a confirmed/suspected case of COVID-19 the previous 2 weeks before the symptom onset);
- Possible (symptoms consistent with COVID-19, with negative RT-PCR and/or antibody tests, with/without radiological signs but WITHOUT contact with a confirmed or suspected case of COVID-19 the previous 2 weeks before the onset of the symptoms) [18].

This author included two new actors into the equation: the presence of symptoms and the contact with a confirmed/suspected case of COVID-19. Both seem to be conflicting, since almost 40–45% of people infected by the SARS-CoV-2 will be asymptomatic [19]. In addition, the plethora of symptoms produced by COVID-19 could also lead to confusion. For instance, the most common symptoms experienced by patients with COVID-19, i.e., fever, fatigue, dyspnea, or productive cough [20], are also usually reported in other respiratory infections, e.g., community-acquired pneumonia [21]. Additionally, patients with COVID-19 also exhibit non-respiratory symptoms, e.g., neurological symptoms (i.e., headaches, anosmia or ageusia), gastrointestinal symptoms (i.e., diarrhea or vomiting) or dermatological disorders (i.e., hair loss, skin rashes) [22]. Therefore, “probable” and “possible” categories (based on symptoms “consistent” with COVID-19) seem to be potentially broad and could include people suffering from respiratory infections but not COVID-19. Similarly, the fact that a person has contact with a confirmed/suspected case of COVID-19 does not confirm that this individual will be infected, although it is clear that individuals who have unprotected contact with infectious subjects have a significant increase in symptoms [23]. Similarly, if a contact develops potential symptoms, those symptoms experienced by this subject (if they ever appear) could be a consequence of stress due to exposure and not just are due to SARS-CoV-2 infection.

Thus, we propose that determining the prevalence of post-COVID symptoms should be based on two main premises: 1, there should be a temporal relationship between the symptoms and COVID-19; and 2, symptoms should appear after the SARS-CoV-2 infection (new post-COVID symptoms, not being previously present). These premises are highly important for future research since symptoms usually seen in “long-haulers” can also be present in the general population which has been exposed to other infectious agents or to a catastrophic situation, like the current one, and be mostly related to lockdown, unemployment, anxiety, fear, social alarm, or others. Including these “possible” and “probable” cases in current research on post-COVID symptoms could add heterogeneity and uncertainty to these studies and thus obstruct the generation of future evidence and limit the utility of therapeutic trials. Hence, clinicians and researchers must ascertain if the symptoms are related (or not) to a potential SARS-CoV-2 infection [24]. Nevertheless, it is important to consider that all diagnostic procedures, including RT-PCR, COVID-19 antibody tests or radiological imaging, have limitations. For instance, radiological studies (CT scans) are more sensitive than RT-PCR, but they have low specificity [25]. Similarly, the sensitivity of antibody testing is also low during the first week from symptom onset, but it could be used as a complement to other diagnostic procedures [26].

Based on these premises, to determine if post-COVID symptoms are mainly caused by COVID-19, they should appear after a positive diagnosis of SARS-CoV-2 infection (timeframe). However, a prevailing problem is that millions of people can also be infected by COVID-19 but never receive a positive COVID-19 diagnosis due to the sanitary situation at a particular time. The current challenge would be to identify those individuals developing potential post-COVID symptoms but without a positive diagnosis, i.e., what Raveendran denominated “probable” or “possible” long COVID [18].

## 3. Is There a Timeframe for Defining Post-COVID Symptoms?

One of the most important topics that should be clarified is the timeframe used for the definition of post-COVID symptoms. Baig proposed that symptoms presenting beyond 3 weeks after the SARS-CoV-2 infection should be considered prolonged or persistent [27]. Halpin et al. proposed the term post-acute COVID (symptoms beyond 3 weeks) and long post-COVID or persistent chronic post-COVID (symptoms beyond 12 weeks) [28]. The NICE guideline proposes the following classification: acute COVID-19 (symptoms for up to 4 weeks), ongoing symptomatic COVID (symptoms from 4 to 12 weeks), and post-COVID (symptoms developed during or after an infection and continuing for more than 12 weeks) [17]. In this guideline, the term “long COVID” would comprise both subgroups, ongoing symptomatic COVID and post-COVID syndrome [17]. In a subsequent analysis of the NICE guideline, Sivan and Taylor proposed the unified definition of long COVID as “signs and symptoms that continue for more than four weeks and can be attributed to COVID-19 infection” [29].

In this equation, the main issue to consider is: once SARS-CoV-2 infection has been surpassed, when should symptoms experienced by a patient be called post-COVID? First, it should be noted that around 50% of individuals infected with SARS-CoV-2 will exhibit a myriad of symptoms during the infection, the most common being fever, cough, fatigue, dyspnea or cough [20]. Currently, an improvement of symptoms, particularly fever and respiratory manifestations, is usually considered the beginning of the end of the acute phase. It is commonly accepted that acute SARS-CoV-2 infection usually lasts 2–3 weeks, although this timeframe is mainly based on reports about the incubation period [30]. Therefore, the first question that researchers should answer is: when can we confirm that a patient has recovered/is free from SARS-CoV-2 infection and thus consider the presence of post-COVID symptoms? It has been found that SARS-CoV-2 is detectable up to 30 days after the resolution of symptoms in 10–15% of diagnosed individuals, but apparently without potential clinical relevance [31]. However, it has been proposed that this prolonged exposition to SARS-CoV-2 may be one of the underlying mechanisms of the post-COVID symptoms. In fact, current evidence supports the presence of psychologic and neuropsychic symptoms, e.g., anxiety, depression, or sleep disorders, in people who deal with COVID-19 patients, e.g., healthcare professionals, but also in the general population [32,33,34,35]. Therefore, some post-COVID-associated symptoms could also be related to the trauma caused by having contracted a potentially fatal infection. Nevertheless, no longitudinal study investigating the evolution of psychological aspects related to COVID-19 pandemic has been conducted to date. Finally, we should take into account the fact that 50% of individuals infected with SARS-CoV-2 will pass the infection without symptoms [19]. In this large proportion of COVID-19 patients, identification of the infection will be more difficult, and therefore, identifying the development of post-COVID symptoms will be a challenge. In such a scenario, international efforts by developing mobile phone software (e.g., Mawid, Tabaud, Tawakkalna, Sehha, Aarogya setu, TraceTogether, COVID safe, Immuni, or COVID watch) as methods of quick detection of contacts with COVID-19 patients could minimize the number of cases [36].

The second actor to consider is the relapsing/remitting nature of the symptoms since these individuals exhibit a wide range and fluctuations of post-COVID symptoms, with gradual improvement in some, but not in others. For instance, several patients can feel they have recovered from the acute infection, but then days or weeks later, they develop (suddenly or progressively) a plethora of symptoms that can be persisting.

## 4. Integrative Classification for Defining Post-COVID Symptoms

Based on the above considerations, we would like to propose the following classification for post-COVID symptoms (pending of validation in future studies). This categorization should be taken as a dynamic and complex process that may integrate potential biological, psychological, and social factors which could predispose or promote the development of post-COVID symptoms. This classification is also conditioned by whether a patient has required hospital admission or not.

- Transition Phase: Symptoms potentially associated with acute COVID-19: symptoms up to 4–5 weeks;
- Phase 1: Acute post-COVID symptoms: symptoms from week 5 to week 12;
- Phase 2: Long post-COVID symptoms: symptoms from week 12 to week 24;
- Phase 3: Persistent post-COVID symptoms: symptoms lasting more than 24 weeks.

**Figure 1:**
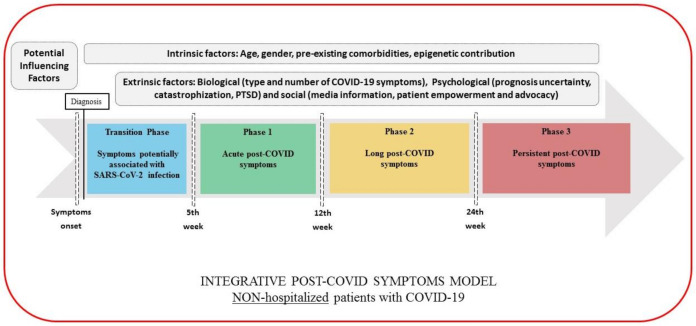
Integrative post-COVID symptoms model in non-hospitalized patients showing transition phase (blue), and phases 1 (green), 2 (yellow), and 3 (red) of post-COVID symptoms. PTSD: post-traumatic stress disorder.

**Figure 2:**
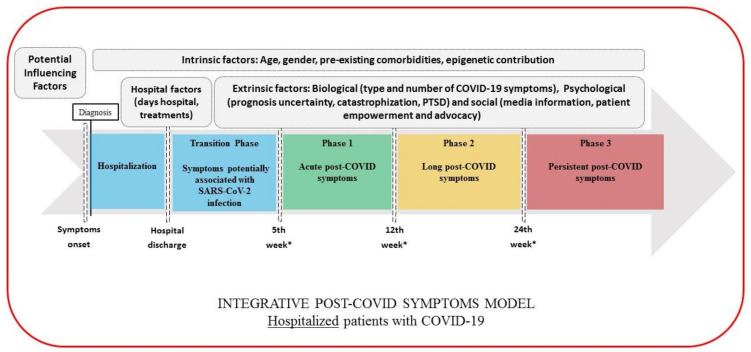
Integrative post-COVID symptoms model in hospitalized patients showing transition phase (blue), and phases 1 (green), 2 (yellow), and 3 (red) of post-COVID symptoms. PTSD: post-traumatic stress disorder.

**Figure 3:**
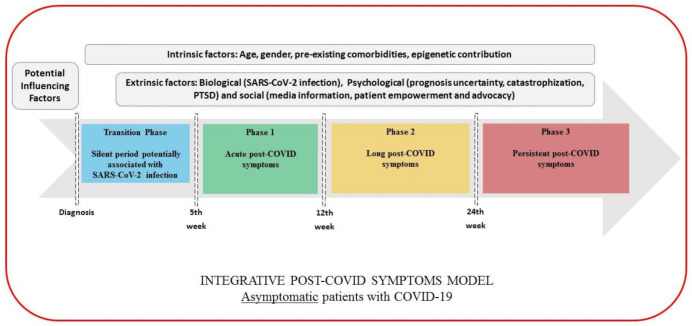
Integrative post-COVID symptoms model in asymptomatic individuals showing transition phase (blue), and phases 1 (green), 2 (yellow), and 3 (red) of post-COVID symptoms. PTSD: post-traumatic stress disorder.

The figures graph the classification proposed where the time reference point is different depending on whether we are speaking of non-hospitalized (onset of the symptoms, Figure 1), hospitalized (hospital discharge, Figure 2) or asymptomatic (only positive testing for COVID-19, Figure 3) patients, and considering the presence of intrinsic (age, gender, pre-existing comorbidities, epigenetics) and extrinsic factors (biological, psychological, and social), but also hospital factors (days at hospital, Intensive Care Unit (ICU) admission or not, prolonged bedding, adverse events derived from interventions).

As can be seen, in the transition phase, symptoms should be cautiously interpreted as “potentially” related to the SARS-CoV-2 infection before being considered post-COVID symptoms. In order to increase the specificity of the definition of post-COVID symptoms, potential sequelae related to hospitalization (i.e., prolonged bedding, ventilator related barotrauma, critical illness polyneuropathies, etc.) should be first ruled out before establishing the diagnosis. Thus, we also propose a transition phase after hospitalization (4–5 weeks after discharge) as a “wash out” period to better define actually related acute post-COVID symptoms. The duration of this phase will depend on the hospitalization time (days, weeks, months) and the “wash out” period.

In this integrative model, we also propose the distinction of “long” (from week 12 to week 24) and “persistent” (from week 24 on) in order to distinguish symptoms with a delayed but progressive improvement, from actual “persistent” symptoms which may have a protracted and refractory course, less probability of spontaneous resolution, and a higher necessity of a multidisciplinary approach.

All these interactions support the proposal of the review by the National Institute for Health Research (NIHR) [37], which has suggested that people experiencing long COVID may exhibit different syndromes, such as post-intensive care syndrome (equivalent to the acute post-COVID phase), post-viral fatigue syndrome (if fatigue is the predominant post-COVID symptom), permanent organ damage (an underlying mechanism explaining long-term symptoms), and long-term COVID syndrome (equivalent to long and persistent post-COVID phases) based on the premise that post-COVID symptoms range in intensity and duration and are not linear or sequential [38].

## 5. Conclusions

This commentary proposes a definition of post-COVID symptoms when they appear after a confirmed diagnosis of SARS-CoV-2 infection; however, some exceptions can appear since not all people infected by SARS-CoV-2 receive a positive diagnosis. Based on the relapsing/remitting nature of post-COVID symptoms, we proposed an integrative classification integrating potential intrinsic and extrinsic factors and hospitalization data which could promote post-COVID symptoms. The most important topic is to establish the time reference points for defining persistent post-COVID symptom. The current discussion should be considered in future studies.

## Data Availability

Not applicable.

## References

[1] Marshall M. (2020). The lasting misery of coronavirus long-haulers. Nature.

[2] Rubin R. (2020). As their numbers grow, COVID-19 “Long Haulers” stump experts. JAMA.

[3] No authors listed (2020). Long COVID: Let patients help define long-lasting COVID symptoms. Nature.

[4] Callard F., Perego E. (2021). How and why patients made Long Covid. Soc. Sci. Med..

[5] The Lancet (2020). Facing up to long COVID. Lancet.

[6] Carfì A., Bernabei R., Landi F., Gemelli against COVID-19 Post-Acute Care Study Group (2020). Persistent Symptoms in Patients after Acute COVID-19. JAMA.

[7] Garrigues E., Janvier P., Kherabi Y., Le Bot A., Hamon A., Gouze H., Doucet L., Berkani S., Oliosi E., Mallart E. (2020). Post-discharge persistent symptoms and health-related quality of life after hospitalization for COVID-19. J. Infect..

[8] Carvalho-Schneider C., Laurent E., Lemaignen A., Beaufils E., Bourbao-Tournois C., Laribi S., Flament T., Ferreira-Maldent N., Bruyère F., Stefic K. (2021). Follow-up of adults with noncritical COVID-19 two months after symptom onset. Clin. Microbiol. Infect..

[9] Arnold D.T., Hamilton F.W., Milne A., Morley A.J., Viner J., Attwood M., Noel A., Gunning S., Hatrick J., Hamilton S. (2020). Patient outcomes after hospitalisation with COVID-19 and implications for follow-up: Results from a prospective UK cohort. Thorax.

[10] Mandal S., Barnett J., Brill S.E., Brown J.S., Denneny E.K., Hare S.S., Heightman M., Hillman T.E., Jacob J., Jarvis H.C. (2020). ‘Long-COVID’: A cross-sectional study of persisting symptoms, biomarker and imaging abnormalities following hospitalisation for COVID-19. Thorax.

[11] Nehme M., Braillard O., Alcoba G., Aebischer-Perone S., Courvoisier D., Chappuis F., Guessous I. (2020). COVID-19 symptoms: Longitudinal evolution and persistence in outpatient settings. Ann. Intern. Med..

[12] Tenforde M.W., Kim S.S., Lindsell C.J., Rose E.B., Shapiro N.I., Files D.C., Gibbs K.W., Erickson H.L., Steingrub J.S., Smithline H.A. (2020). Symptom duration and risk factors for delayed return to usual health among outpatients with Covid-19 in a multistate health care systems network-United States, March–June 2020. MMWR Morb. Mortal. Wkly. Rep..

[13] Stavem K., Ghanima W., Olsen M.K., Gilboe H.M., Einvik G. (2020). Persistent symptoms 1.5–6 months after COVID-19 in non-hospitalised subjects: A population-based cohort study. Thorax.

[14] Page M. (2018). The British Medical Association Illustrated Medical Dictionary.

[15] Mahase E. (2020). COVID-19: What do we know about “long COVID”?. BMJ.

[16] Nabavi N. (2020). Long COVID: How to define it and how to manage it. BMJ.

[17] National Institute for Health and Care Excellence (NICE), Royal College of General Practitioners, Healthcare Improvement Scotland SIGN (2020). COVID-19 Rapid Guideline: Managing the Long-Term Effects of COVID-19.

[18] Raveendran A. (2020). Long COVID-19: Challenges in the diagnosis and proposed diagnostic criteria. Diabetes Metab. Syndr..

[19] Oran D.P., Topol E.J. (2020). Prevalence of asymptomatic SARS-CoV-2 Infection: A narrative review. Ann. Intern. Med..

[20] Alimohamadi Y., Sepandi M., Taghdir M., Hosamirudsari H. (2020). Determine the most common clinical symptoms in COVID-19 patients: A systematic review and meta-analysis. J. Prev. Med. Hyg..

[21] Ticona J.H., Zaccone V.M., McFarlane I.M. (2021). Community-acquired pneumonia: A focused review. Am. J. Med. Case Rep..

[22] Tostmann A., Bradley J., Bousema T., Yiek W.-K., Holwerda M., Bleeker-Rovers C., Oever J.T., Meijer C., Rahamat-Langendoen J., Hopman J. (2020). Strong associations and moderate predictive value of early symptoms for SARS-CoV-2 test positivity among healthcare workers, the Netherlands, March 2020. Eurosurveillance.

[23] Magnavita N., Tripepi G., Di Prinzio R.R. (2020). Symptoms in health care workers during the COVID-19 epidemic. A cross-sectional survey. Int. J. Environ. Res. Public Health.

[24] Yelin D., Margalit I., Yahav D., Runold M., Bruchfeld J. (2020). Long COVID-19-it’s not over until?. Clin. Microbiol. Infect..

[25] Mair M.D.D., Hussain M., Siddiqui S., Das S., Baker A., Conboy P., Valsamakis T., Uddin J., Rea P. (2021). A systematic review and meta-analysis comparing the diagnostic accuracy of initial RT-PCR and CT scan in suspected COVID-19 patients. Br. J. Radiol..

[26] Deeks J.J., Dinnes J., Takwoingi Y., Davenport C., Spijker R., Taylor-Phillips S., Adriano A., Beese S., Dretzke J., Di Ruffano L.F. (2020). Antibody tests for identification of current and past infection with SARS-CoV-2. Cochrane Database Syst. Rev..

[27] Baig A.M. (2020). Chronic COVID Syndrome: Need for an appropriate medical terminology for Long-COVID and COVID Long-Haulers. J. Med. Virol.

[28] Halpin S., O’Connor R., Sivan M. (2020). Long COVID and chronic COVID syndromes. J. Med. Virol.

[29] Sivan M., Taylor S. (2020). NICE guideline on long covid. BMJ.

[30] Lauer S.A., Grantz K.H., Bi Q., Jones F.K., Zheng Q., Meredith H.R., Azman A.S., Reich N.G., Lessler J. (2020). The incubation period of coronavirus disease 2019 (COVID-19) from publicly reported confirmed cases: Estimation and application. Ann. Intern. Med..

[31] Ikegami S., Benirschke R., Flanagan T., Tanna N., Klein T., Elue R., Debosz P., Mallek J., Wright G., Guariglia P. (2020). Persistence of SARS-CoV-2 nasopharyngeal swab PCR positivity in COVID-19 convalescent plasma donors. Transfusion.

[32] Magnavita N., Soave P.M., Ricciardi W., Antonelli M. (2020). Occupational stress and mental health of anesthetists during the COVID-19 pandemic. Int. J. Environ. Res. Public Health.

[33] Huang Y., Zhao N. (2020). Generalized anxiety disorder, depressive symptoms and sleep quality during COVID-19 outbreak in China: A web-based cross-sectional survey. Psychiatry Res..

[34] Vindegaard N., Benros M.E. (2020). COVID-19 pandemic and mental health consequences: Systematic review of the current evidence. Brain Behav. Immun..

[35] Magnavita N., Di Prinzio R.R., Chirico F., Sacco A., Quintavalle G. (2020). COVID-19 and staff mental health: Is there an evidence? An Italian field study. Eur. J. Public Health.

[36] Alanzi T. (2021). A Review of mobile applications available in the app and google play stores used during the COVID-19 outbreak. J. Multidiscip. Healthc..

[37] NIHR Living with Covid-19. A Dynamic Review of the Evidence around Ongoing COVID-19 SYMPTOMS (Often Called Long COVID). https://evidence.nihr.ac.uk/themedreview/living-with-covid19.

[38] Mahase E. (2020). Long COVID Could Be Four Different Syndromes, Review Suggests. BMJ.

